# Relationship between the Condylion–Gonion–Menton Angle and Dentoalveolar Heights

**DOI:** 10.3390/ijerph17093309

**Published:** 2020-05-09

**Authors:** Rosa Valletta, Roberto Rongo, Ada Carolina Pango Madariaga, Roberta Baiano, Gianrico Spagnuolo, Vincenzo D’Antò

**Affiliations:** 1 Department of Neuroscience, Reproductive Sciences and Oral Sciences, University of Naples “Federico II”, 80138 Naples, Italy; valletta@unina.it (R.V.); roberto.rongo@gmail.com (R.R.); apangom@gmail.com (A.C.P.M.); baiano.roberta89@gmail.com (R.B.); vincenzo.danto@unina.it (V.D.); 2 Medicine, Surgery and Dentistry “Schola Medica Salernitana” Department, University of Salerno, 84084 Fisciano Salerno, Italy

**Keywords:** dentoalveolar heights, mandibular divergency, facial growth, cephalometric analysis

## Abstract

An accurate estimation of both facial growth and the dentoalveolar dimension is key to successful treatment. The aim of this study was to analyze the relation between the Condylion–Gonion–Menton angle (CoGoMe^) and dentoalveolar heights in a population of patients from southern Italy. This retrospective study analyzed 270 cephalograms of 115 males (42.1%, mean age 15.5 ± 5.2 years) and 155 females (57.9%, mean age 15.6 ± 5.9 years). The facial divergency was evaluated with the Sella–Nasion and Gonion–Gnation angle (SN^GoGn), mandibular structure with the CoGoMe^, and dentoalveolar heights were assessed in four measurements: upper anterior (UADH), lower anterior (LADH), upper posterior (UPDH), and lower posterior (LPDH). Data were analyzed by means of Pearson’s correlation and linear regression model (*p* < 0.05). All the dentoalveolar heights were strongly correlated among them (*p* < 0.001). The UADH was correlated with the SN^GoGn (r = 0.145; *p* = 0.017), while the LPDH was correlated with the CoGoMe^ (r = −0.183; *p* = 0.003). Moreover, there was a positive association between the UADH and the SN^GoGn (B = 0.08; 95% CI: 0.014–0.144; *p* = 0.017), and a negative association between the CoGoMe^ and the LPDH (B = −0.098; 95% CI: −0.161–0.035; *p* = 0.003). Facial divergency and mandibular structure are associated with dentoalveolar heights.

## 1. Introduction

Orthodontists have long been interested in the difference in the diagnosis, treatment planning, and treatment responses between hyperdivergent and hypodivergent facial typologies, and an accurate estimation of dentoalveolar dimension is key to successful treatment [1]. The mandibular growth pattern can affect several aspects of the orthodontic treatment plan such as the decision of extraction, the anchorage choice, the orthodontic biomechanics, and the type and period of retention [2]. The vertical development of the facial skeleton during childhood and adolescence has been described in detail by Bjork [3] and Bjork and Skieller [4]. Mandibular growth, which is mainly related to condylar growth, was distinguished in forward and backward rotations and these were characterized by seven clinical signs [5]. Mandibular growth rotation is also used to identify different facial typologies, classified as dolichofacial, normofacial, and brachyfacial [6]. A dolichofacial typology shows excessive vertical facial growth, a backward rotation of the mandible, a labial incompetence tendency, and it is usually associated with an increased Sella–Nasion^Gonion–Gnathion (SN^GoGn) angle and an increased maxillary/mandibular plane angle (AnsPns^GoGn). A brachyfacial typology has reduced vertical facial growth, a forward mandibular rotation, and it is usually accompanied by a reduced SN^GoGn and a reduced AnsPns^GoGn [2,7,8]. Both condylar growth and dentoalveolar development play a crucial role in the evolution of the facial skeleton; indeed, differential growth in these structures is particularly influential with regard to the vertical development of facial characteristics [9]. The dentoalveolar segment develops with the eruption of teeth and is composed of the teeth and the surrounding alveolar bone. When the teeth of the opposing jaws come in contact with each other, they establish the dentoalveolar heights [10]. Several studies have shown that the dentoalveolar bone grows and changes throughout life [11,12]. The alveolar structure and teeth form the functional components of the jaws, participate in the occlusal dynamics, and have a role in establishing sagittal and vertical maxillomandibular relationships. Indeed, they may also play the compensatory function of camouflaging skeletal deviations between the jaws [13,14]. Dentoalveolar compensation has two main components. The first is related to the vertical development of the basal bone and dentoalveolar heights, while the second affects incisor inclination [15,16]. Several authors have investigated the relationship between dentoalveolar heights and different facial typologies [1,4,10,17,18,19,20,21,22], and have reported contrasting results. According to some studies, hyperdivergent patients might present a greater upper anterior dental height (UADH; distance from the upper central incisor edge to the palatal plane) and/or lower anterior dental heights (LADH; distance from the lower central incisor edge to the mandibular plane) and/or a smaller upper (UPDH; distance from the mesiobuccal cusp tip of the maxillary first molar to the palatal plane) and/or lower posterior dentoalveolar heights (LPDH; distance from the mesiobuccal cusp tip of the mandibular first molar to the mandibular plane) compared with normodivergent or hypodivergent patients [1,19,23,24]. On the other hand, other studies found that all dentoalveolar heights were significantly greater in patients with a hyperdivergent mandibular growth pattern as opposed to those with a normodivergent growth pattern [18,25,26,27]. A recent study found that among vertical growers only the anterior dental heights (UADH and LADH) were significantly increased when compared with normal and horizontal growers, while posterior dental heights (UPDH and UADH) were not significantly different among vertical patterns [1]. Finally, Martina et al. reported that the amount of posterior dentoalveolar heights decreases when the mandibular plane angle increases and when the lower facial height increases [21]. Hence, it is not clear what the relationship is between the mandibular growth pattern and dentoalveolar heights. This overwhelming contrast in the results of the studies may be due to the different sample selection criteria, but the role of genetic and environmental factors cannot be ignored [28,29]. One of the morphological characteristics of the lower jaw related to anterior/posterior growth patterns is the angle formed by the condylar axis and the mandibular base [30]. As discovered by Franchi et al., the Condylion–Gonion–Menton angle (CoGoMe^) is able to recognize and distinguish the responsiveness of patients to Class II functional treatment [31]. A recent investigation of the CoGoMe^ in a population of patients from southern Italy found that it was associated with mandibular divergency and can be considered a predictor of vertical growth patterns, with a mean value for normodivergent patients of 127.1 ± 6.1 [32]. Studying the relationship between this angle and the vertical characteristics of the dentoalveolar bone may offer orthodontists a valid cephalometric instrument for predicting a patient’s growth and improving the treatment quality and prognosis. The aim of this study was to analyze the relation between the CoGoMe^ and dentoalveolar heights in a population of patients from southern Italy. The null hypothesis was that there is no relationship between the CoGoMe^ and dentoalveolar heights.

## 2. Materials and Methods

### 2.1. Subjects

This research protocol was approved by the Ethics Committee of the University of Naples Federico II (121/19). For this retrospective study, the cephalograms of 270 subjects (115 males (42.1%; mean age 15.5 ± 5.2 years) and 155 females (57.9%; mean age 15.6 ± 5.9 years)) from 8–53-years-old were selected by a database of the Section of Orthodontics of the University of Naples “Federico II” (Table 1).

The cephalograms were selected based on the following inclusion criteria:

- Caucasian race,
- age ≥ 8, and
- a good quality lateral X-ray.

The following conditions were considered further exclusion criteria:

- patients with systemic diseases,
- patients with genetic syndromes,
- previous orthodontic treatment, and
- a history of obstructed nose breathing.

### 2.2. Cephalometric Evaluation

**Figure 1:**
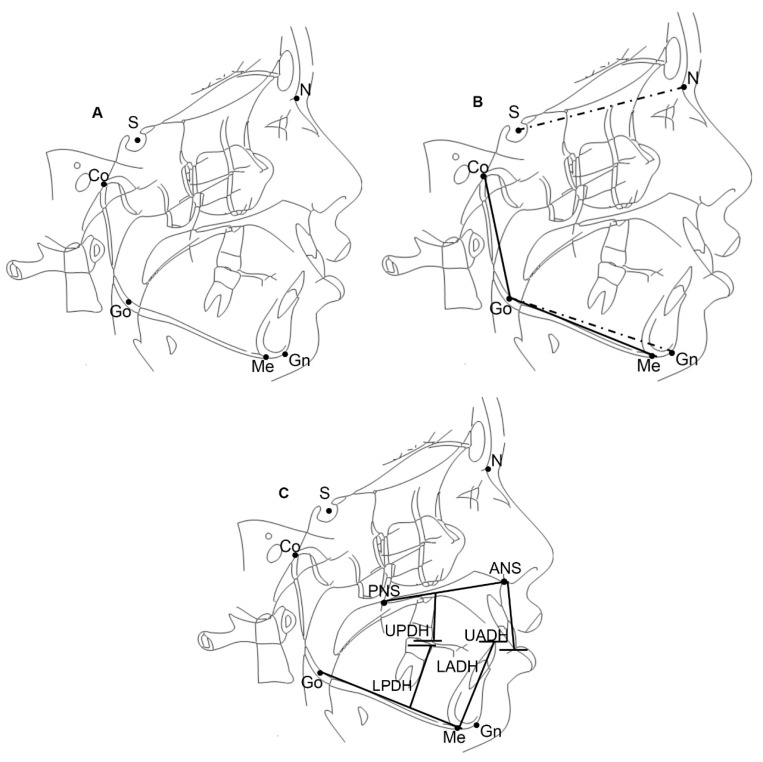
Skeletal landmarks, lines, and measurements used for cephalometric analysis. (**A**) **Landmarks and lines**: Sellion (S), the midpoint of the cavity of sella turcica; Nasion (N), the anterior point of the intersection between the nasal and frontal bones; Menton (Me), the intersection point of the posterior symphysis contour and the inferior contour of the corpus; Gonion (Go), the point on the contour of the mandible determined by bisecting the angle formed by the mandibular and ramus plane; Gnation (Gn), the point of intersection between the contour of the chin and the line bisecting the inferior border of the mandible and the line passing through N and Pg; Condylion (Co), the most posterior and superior points on the mandibular condyle; Anterior Nasal Spine point (ANS), the most anterior point of the bony hard palate in the mid-sagittal plane; Posterior Nasal Spine point (PNS), the most posterior point of the bony hard palate in the mid-sagittal plane; (**B**) **Plane and angular measurements**: Palatal plane (PP), a line that connects ANS to PNS; Mandibular plane (MP), a line that connects Go to Me; Gonion Gnation plane (GoGn), a line that connects Go to Gn; Nasion Sellion plane (SN), a line that connects N to S; Condilion Gonion Plane (CoGo), a line that connects Co to Go. SN^GoGn, the angle between the SN plane and GoGn; CoGoMe^, the angle between the CoGo and GoMe. (**C**) **Vertical measurements**: Dentoalveolar heights were measured for maxillary and mandibular incisors and first molars as the upper anterior dental height (UADH) measured as the perpendicular distance from the maxillary incisor edge to the palatal plane (mm); lower anterior dental height (LADH) measured as the perpendicular distance from the mandibular central incisor edge to the mandibular plane (mm); upper posterior dental height (UPDH) measured as the perpendicular distance from the mesiobuccal cusp tip of the maxillary first molar to the palatal plane (mm); and lower posterior dental height (LPDH) measured as the perpendicular distance from the mesiobuccal cusp tip of the mandibular first molar to the mandibular plane (mm).

All lateral radiographs were taken in natural head position before the orthodontic treatment [33,34,35]. One operator traced all lateral cephalograms with cephalometric software (Delta-Dent, Outside Format, Piolla, Italy). Cephalometric landmarks, lines, and measurements identified and traced on lateral cephalograms in order to evaluate facial typology are shown in Figure 1A–C. To determine facial divergency and sagittal malocclusion the angles SN^GoGn and ANPg^ (Point A-Nasion-Pogonion angle) were used. The sagittal malocclusion was classified into three groups according to the ANPg^: Class III with an ANPg^ less than 0°, Class I with an ANPg^ between 0° and 4°, and Class II with an ANPg^ greater than 4°. Subjects were classified as brachyfacial when SN^GoGn was less than 27°, normofacial when SN^GoGn was between 27° and 37°, and dolichofacial when SN^GoGn was greater than 37°. The mandibular structure was measured with the CoGoMe^, which is the angle between the condylar axis (Condylion–Gonion) and the mandibular base (Gonion–Menton). Finally, the dentoalveolar heights (upper or lower and anterior or posterior) were measured as

- UADH: upper anterior dental height measured as the perpendicular distance from the maxillary incisor edge to the palatal plane (mm),
- LADH: lower anterior dental height measured as the perpendicular distance from the mandibular central incisor edge to the mandibular plane (mm),
- UPDH: upper posterior dental height measured as the perpendicular distance from the mesiobuccal cusp tip of the maxillary first molar to the palatal plane (mm), and
- LPDH: lower posterior dental height measured as the perpendicular distance from the mesiobuccal cusp tip of the mandibular first molar to the mandibular plane (mm).

### 2.3. Statistical Analysis

Sample size calculation was performed, and it was found that a sample size of 218 achieved 90% power to detect an effect size (f²) of 0.050 attributable to one independent variable(s) using an F-test with a significance level (alpha) of 0.050. The variables tested were adjusted for an additional two independent variable(s). The calculations assume an unconditional (random X’s) model.

The method error was calculated from 101 randomly selected lateral cephalograms using Dahlberg’s formula and the paired Student’s *t*-test with the type I error set at 0.05 [20]. Categorical variables were reported as frequencies and percentages, and continuous variables were reported as means and standard deviations. The Shapiro–Wilk (SW) test was used to evaluate normal distribution of the data. Pearson’s correlation analysis was used to assess the relationship among dentoalveolar heights, and between each height and each skeletal cephalometric variable. Linear regression analysis was performed to evaluate whether there was an association between dental heights and the CoGoMe^ or SN^GoGn (used as independent variable). For this analysis two models were performed unadjusted and adjusted for age and sex. Beta coefficients and 95% confidence intervals (95% CI) were calculated. The sample was further divided according to the age (Growing ≤16 years old and Not Growing >16 years old) and the same correlation analysis and regression model were performed. All statistical tests were two-sided. *p*-values less than 0.05 were considered significant. The Standard Statistical Software Package (SPSS version 22.0, SPSS, IBM, Armonk, NY, USA) was used for statistical analysis.

## 3. Results

The method error for linear dentoalveolar heights was between 0.12 and 0.65; the method error for angular measurement was 0.64 for the SN^GoGn and 0.57 for the CoGoMe^.

There was no systematic error for any measurements (Student’s *t*-test, *p* < 0.05). The sample included 50 Class III, 89 Class II, and 131 Class I subjects, moreover 54 subjects were brachyfacial, 57 dolichofacial, and 159 normofacial. The mean values for the CoGoMe^ and the dentoalveolar heights are given in Table 2. Pearson’s correlation analysis (Table 3) showed that all the dentoalveolar heights were strongly correlated each other (*p* < 0.001), and the SN^GoGn and CoGoMe^ were strongly correlated (r = 0.656; *p* < 0.001). Finally, the UADH was correlated with the SN^GoGn (r = 0.145; *p* = 0.017), while the LPDH was correlated with the CoGoMe^ (r= −0.183; *p* = 0.003).

The results of the regression analysis (Table 4 and Table 5) showed a positive association between the UADH and the SN^GoGn (B = 0.08; 95% CI: 0.014–0.144; *p* = 0.017), and a negative association between the CoGoMe^ and the LPDH (B = −0.098; 95% CI: −0.161–0.035; *p* = 0.003). When the sample was divided in Growing ≤16 years old and Not Growing >16 years old subjects both the correlation and the regression analyses showed similar results than the analysis of the whole sample.

## 4. Discussion

In this study we wanted to assess the association between mandibular growth and the dentoalveolar heights. We found a negative association between the CoGoMe^ and the LPDH and a positive association between the SN^GoGn and the UADH, while all the dentoalveolar heights were positively correlated among them. It is still not clear if facial height is genetically determined or if it is more related to the eruption of teeth during growth, or even during adulthood, which if excessive may result in the increase in facial height [36,37,38]. In fact, dentoalveolar heights have the innate ability to adapt to the underlying developing or established skeletal dysplasia [13,15,27,36,39]. This process is known as dentoalveolar compensation [13,15,36,39]. Our sample showed a positive correlation between all the dentoalveolar heights, that increased all together, which is consistent with the normal development of the stomatognathic system [36,37,40]. The CoGoMe^ is an angle that can be used to evaluate the morphological characteristics of the lower jaw related to the anterior/posterior growth pattern; when it is increased, the patient usually has excessive vertical facial growth, backward rotation of the mandible and a reduction in lower posterior dentoalveolar heights (LPDH) [32,41]. According to Enlow and his growth theory, the mandibular ramus positions and dimensions affect the lower arch in occlusion with the maxillary arch [42]. Consequently, dentoalveolar heights adapt to the mandibular growth, reducing the LPDH. This may explain why we found a reduction of the LPDH in relation to a negative association with the CoGoMe^. The SN^GoGn is a very useful diagnostic parameter to consider before starting an orthodontic treatment because it evaluates the facial pattern of a subject and reflects the variability of the mandibular plane in relation to the anterior cranial base [43]. In fact, it is considered a good, reliable indicator when assessing vertical growth pattern because its results are increased in hyperdivergent patients. In our study, each degree of increase in the SN^GoGn resulted in an increase in the upper anterior dentoalveolar heights (UADH) by 0.08 mm. Therefore, the anterior incisal extrusion tends to compensate for the mandibular clockwise rotation, and these findings are in accordance with previous authors [1,18,19,21].

Indeed, Zafar in his study showed a significant increase in the UADH in the hyperdivergent group compared with both the normodivergent and hypodivergent groups [10] and other studies have found that a hypodivergent mandibular growth pattern is associated with decreased dentoalveolar heights [1,10,18,23,44]. In contrast, Betzenberger et al. showed that the hyperdivergent subjects have decreased dentoalveolar heights when compared with the normodivergent subjects. However, Betzenberger investigated the same features in growing patients and adults and found that age affects dentoalveolar heights. Indeed, dentoalveolar compensation was accomplished by relative increases in maxillary and mandibular anterior dentoalveolar heights in the mixed dentition group and by relative decreases in maxillary and mandibular posterior dentoalveolar heights in the permanent dentition group [19]. As previously mentioned, these contrasting results may be due to genetic factors [28]. Nevertheless, other factors related to the inclusion criteria and cephalometric analysis must be considered. For example, many studies included different ethnicities and came up with variable results. Furthermore, several studies in the literature distinguish only upper and lower dentoalveolar heights without addressing the anterior and posterior measurements [17,21]. Finally, another important factor to be considered is that the CoGoMe^ was not previously assessed in association with dentoalveolar heights. The CoGoMe^ is a variable related prevalently to mandibular structure (condylar axis and mandibular base); hence its evaluation is not affected by any other external structures [32]. A greater CoGoMe^ is strongly related to a short ramus height and a steeper mandibular plane. However, not all patients with an increased CoGoMe^ presented an increased SN^GoGn. Similarly, the SN^GoGn is also affected by the position of the Nasion; if the Nasion is very high and backward it can simulate a hyperdivergent facial type, with a normal mandibular structure. An increase in the SN^GoGn is not necessarily indicative of a hyperdivergent pattern; the tendency toward a backward rotation in fact may be due to environmental influences leading to abnormal habits, such as mouth breathing, tongue thrusting, and altered head position. Hence, in some cases the increases in the SN^GoGn are more related to a positional modification of the mandible, without a short ramus, that does not influence the LPDH but stimulates a greater growth of the UADH [45]. On the contrary, according to our study, the CoGoMe^, which is closely attached to the morphology of the facial ramus, has a greater influence on the LPDH. Therefore, this means that when diagnosing a hyperdivergent patient, even a structural evaluation of the jaw cannot be disregarded. Finally, the evaluation of dentoalveolar heights may have a fundamental role in the treatment planning. For example, in patients with an increased CoGoMe^ where the LADH are reduced, to avoid any overcompensation of the LADH, it might be more indicated to reduce the UADH to promote the mandibular anterotation [46].

This study presents some limitations due to the cross-sectional study design, such that the age range was wide. Even if statistically evaluated, there could be an effect of growth on the dental compensation. In addition, there was no data on patients’ oral habits. On the other hand, this study also presents some strengths, such that it was the first study that evaluated the association between dentoalveolar heights and the CoGoMe^. Moreover, the sample size was relatively wide, which allowed us to achieve a more reliable result and a good estimation of the data. Further studies are necessary to evaluate these cephalometric parameters and their influence on facial growth.

## 5. Conclusions

The evaluation of the dentoalveolar heights is an important consideration in orthodontic treatment planning to fulfill the objectives of the treatment. Understanding the relationship between dentoalveolar heights and mandibular divergency may suggest to the clinician how modify the teeth position to correct or to compensate vertical skeletal discrepancy, avoiding possible overcompensation. The analysis identified that all the dentoalveolar heights were correlated among them, while the UADH showed a positive association with the SN^GoGn and the LPDH showed a negative association with the CoGoMe^. Dentoalveolar heights are associated with the mandibular growth pattern.

## Figures and Tables

**Table 1:** Age and gender distribution.

Variables	*N*	Mean ± SD	95% CI
Age	270	15.6 ± 5.9	14.9–16.3
**Gender**	***N***	**Percentage %**	
Male	115	15.5 ±5.2	14.5–16.5
Female	155	15.7 ± 6.4	14.7–16.7

Data are presented as mean ± standard deviation (SD) and 95% confidence interval (95% CI).

**Table 2:** Descriptive statistics of the assessed variables.

Variables	Mean ± SD	95% CI
**CoGoMe^**	127.1 ± 7.7	126.2, 128.0
**SN^GoGn**	31.9 ± 6.8	31.1, 32.7
**UADH**	28.6 ± 3.7	28.1, 29.0
**LADH**	39.3 ± 4.7	38.7, 39.8
**UPDH**	22.4 ± 3.6	22.0, 22.8
**LPDH**	29.0 ± 4.1	28.5, 29.5

Data are presented as mean ± standard deviation (SD) and 95% confidence interval (95% CI). CoGoMe^: the angle between the CoGo and GoMe. SN^GoGn: the angle between the SN plane and GoGn; (C) UADH: Upper anterior dental height (mm); LADH: Lower anterior dental height (mm) UPDH: Upper posterior dental height (mm) LPDH: lower posterior dental height (mm).

**Table 3:** Pearson correlation analysis.

Number of Subjects		CoGoMe^		UADH		LADH		UPDH		LPDH	
		t	*p*-Value	t	*p*-Value	t	*p*-Value	t	*p*-Value	t	*p*-Value
**Total (270)**	**SN^GoGn**	**0.656**	**<0.001**	**0.145**	**0.017**	0.006	0.928	−0.015	0.807	−0.078	0.201
	**CoGoMe^**			−0.021	0.733	−0.118	0.053	−0.102	0.095	**−0.183**	**0.003**
	**UADH**					**0.774**	**<0.001**	**0.779**	**<0.001**	**0.659**	**<0.001**
	**LADH**							**0.771**	**<0.001**	**0.873**	**<0.001**
	**UPDH**									**0.708**	**<0.001**
**Growing ≤16 years old (194)**	**SN^GoGn**	**0.540**	**<0.001**	**0.202**	**0.005**	0.102	0.159	0.050	0.489	0.032	0.654
	**CoGoMe^**			−0.008	0.912	−0.092	0.200	−0.092	0.201	**−0.143**	**0.007**
	**UADH**					**0.774**	**<0.001**	**0.761**	**<0.001**	**0.629**	**<0.001**
	**LADH**							**0.754**	**<0.001**	**0.867**	**<0.001**
	**UPDH**									**0.650**	**<0.001**
**Not Growing >16 years old (76)**	**SN^GoGn**	**0.770**	**<0.001**	**0.187**	**0.034**	0.008	0.944	0.089	0.444	−0.089	0.443
	**CoGoMe^**			0.083	0.477	−0.008	0.947	0.087	0.457	**−0.148**	**0.006**
	**UADH**					**0.730**	**<0.001**	**0.801**	**<0.001**	**0.654**	**<0.001**
	**LADH**							**0.724**	**<0.001**	**0.840**	**<0.001**
	**UPDH**									**0.682**	**<0.001**

Data are presented as t values and bold text indicates statistically significant value (*p*-value < 0.05).

**Table 4:** Regression analysis between CoGoMeˆ and the different dentoalveolar heights.

		Total (270)			Growing ≤16 years old (194)			Not Growing >16 years old (76)		
Outcome	Model	Beta	95% CI	*p*-Value	Beta	95% CI	*p*-Value	Beta	95% CI	*p*-Value
**UADH**	1 (CoGoMeˆ)	−0.01	−0.07–0.05	0.733	−0.04	−0.08–0.07	0.912	0.03	−0.06–0.13	0.477
	2 (CoGoMeˆ Age Sex)	0.01	−0.04–0.07	0.550						
**LADH**	1 (CoGoMe^)	−0.07	−0.15–0.00	0.053	−0.06	−0.15–0.03	0.200	−0.04	−0.12–0.11	0.947
	2 (CoGoMeˆ Age Sex)	−0.02	−0.09–0.04	0.453						
**UPDH**	1 (CoGoMeˆ)	−0.04	−0.10–0.01	0.095	−0.04	−0.11–0.02	0.201	0.34	−0.06–0.12	0.457
	2 (CoGoMeˆ Age Sex)	−0.01	−0.05–0.05	0.842						
**LPDH**	1 (CoGoMeˆ)	−0.09	−0.16–−0.04	**0.003**	−0.08	−0.16–−0.01	**0.007**	−0.07	−0.14–−0.00	**0.006**
	2 (CoGoMeˆ Age Sex)	−0.07	−0.11–−0.02	**0.004**						

Data are presented as beta and 95% confidence interval (95% CI). Bold text indicates statistically significant value (*p*-value < 0.05). Two different models were proposed: Model 1, CoGoMeˆ and dentoalveolar heights unadjusted comparison (crude Beta); Model 2, Model 1 + age and sex.

**Table 5:** Regression analysis between SN^GoGn and the different dentoalveolar heights.

		Total (270)			Growing ≤16 years old (194)			Not Growing >16 years old (76)		
Outcome	Model	Beta	95% CI	*p*-Value	Beta	95% CI	*p*-Value	Beta	95% CI	*p*-Value
**UADH**	1 (SN^GoGn)	0.08	0.01–0.14	**0.017**	0.12	0.04, 0.21	**0.005**	0.08	0.01–0.17	**0.034**
	2 (SN^GoGn Age Sex)	0.11	0.05–0.17	**0.001**						
**LADH**	1 (SN^GoGn)	−0.01	−0.08–0.08	0.928	0.08	−0.03–0.19	0.159	0.00	−0.12–0.13	0.944
	2 (SN^GoGn Age Sex)	0.05	−0.02–0.13	0.170						
**UPDH**	1 (SN^GoGn)	−0.01	−0.07–0.06	0.807	0.03	−0.05–0.10	0.489	0.04	−0.06–0.13	0.444
	2 (SN^GoGn Age Sex)	0.04	−0.02–0.09	0.205						
**LPDH**	1 (SN^GoGn)	−0.05	−0.12–0.03	0.201	0.02	−0.07–0.11	0.654	−0.04	−0.15–0.07	0.443
	2 (SN^GoGn Age Sex)	−0.00	−0.07–0.06	0.925						

Data are presented as beta and 95% confidence interval (95% CI). Bold text indicates statistically significant value (*p*-value < 0.05). Two different models were proposed: Model 1, SN^GoGn and dentoalveolar heights unadjusted comparison (crude Beta); Model 2, Model 1 + age and sex.

## References

[1] Rasool G., Bibi T. (2016). Comparison of Dentoalveolar Heights in Relation to Vertical Facial Pattern. J. Khyber College Dent..

[2] Schudy F. (1965). The rotation of the mandible resulting from growth: Its implications in orthodontic treatment. Angle Orthod..

[3] Björk A. (1969). Prediction of mandibular growth rotation. Am. J. Orthod..

[4] Björk A., Skieller V. (1972). Facial development and tooth eruption. An implant study at the age of puberty. Am. J. Orthod..

[5] Von Bremen J., Pancherz H. (2005). Association between Björk’s structural signs of mandibular growth rotation and skeletofacial morphology. Angle Orthod..

[6] Tanner A.C.R., Sonis A.L., Lif Holgerson P., Starr J.R., Nunez Y., Kressirer C.A., Paster B.J., Johansson I. (2012). White-spot lesions and gingivitis microbiotas in orthodontic patients. J. Dent. Res..

[7] Riedel R.A. (1952). The relation of maxillary structures to cranium in malocclusion and in normal occlusion. Angle Orthod..

[8] Valletta R., Pango A., Tortora G., Rongo R., Simeon V., Spagnuolo G., D’Antò V. (2019). Association between Gingival Biotype and Facial Typology through Cephalometric Evaluation and Three-Dimensional Facial Scanning. Appl. Sci..

[9] Arat Z.M., Rübendüz M. (2005). Changes in dentoalveolar and facial heights during early and late growth periods: A longitudinal study. Angle Orthod..

[10] Zafar-Ul-Islam S.A., Fida M. (2012). Dentoalveolar heights in skeletal class I normodivergent facial patterns. J. Coll. Physicians Surg. Pak..

[11] Sarnäs K.V., Solow B. (1980). Early adult changes in the skeletal and soft-tissue profile. Eur. J. Orthod..

[12] Tallgren A., Solow B. (1991). Age differences in adult dentoalveolar heights. Eur. J. Orthod..

[13] Ishikawa H., Nakamura S., Iwasaki H., Kitazawa S., Tsukada H., Sato Y. (1999). Dentoalveolar compensation related to variations in sagittal jaw relationships. Angle Orthod..

[14] Rongo R., Bucci R., Adaimo R., Amato M., Martina S., Valletta R., D’anto V. (2020). Two-dimensional versus three-dimensional Fränkel Manoeuvre: A reproducibility study. Eur. J. Orthod..

[15] Anwar N., Fida M. (2009). Compensation for vertical dysplasia and its clinical application. Eur. J. Orthod..

[16] Mangla R., Singh N., Dua V., Padmanabhan P., Khanna M. (2011). Evaluation of mandibular morphology in different facial types. Contemp. Clin. Dent..

[17] Isaacson J.R., Isaacson R.J., Speidel T.M., Worms F.W. (1971). Extreme Variation in Vertical Facial Growth and Associated Variation in Skeletal and Dental Relations. Angle Orthod..

[18] Janson G.R., Metaxas A., Woodside D.G. (1994). Variation in maxillary and mandibular molar and incisor vertical dimension in 12-year-old subjects with excess, normal, and short lower anterior face height. Am. J. Orthod. Dentofac. Orthop..

[19] Betzenberger D., Ruf S., Pancherz H. (1999). The compensatory mechanism in high-angle malocclusions: A comparison of subjects in the mixed and permanent dentition. Angle Orthod..

[20] Enoki C., Telles C.D.S. (2004). Dental-Skeletal Dimensions in Growing Individuals with Variations in the Lower Facial Height. Braz. Dent. J..

[21] Martina R., Farella M., Tagliaferri R., Michelotti A., Quaremba G., van Eijden T.M.G.J. (2005). The Relationship between molar dentoalveolar and craniofacial heights. Angle Orthod..

[22] Islam Z.U., Shaikh A.J., Fida M. (2016). Dentoalveolar Heights in Vertical and Sagittal Facial Patterns. J. Coll. Physicians Surg. Pak..

[23] Opdebeeck H., Bell W. (1978). The short face syndrome. Am. J. Orthod..

[24] Opdebeeck H., Bell W.H., Eisenfeld J., Mishelevich D. (1978). Comparative study between the SFS and LFS rotation as a possible morphogenic mechanism. Am. J. Orthod..

[25] Bell W.H., Creekmore T.D., Alexander R.G. (1977). Surgical correction of the long face syndrome. Am. J. Orthod..

[26] Schendel S.A., Eisenfeld J., Bell W.H., Epker B.N., Mishelevich D.J. (1976). The long face syndrome: Vertical maxillary excess. Am. J. Orthod..

[27] Kucera J., Marek I., Tycova H., Baccetti T. (2011). Molar height and dentoalveolar compensation in adult subjects with skeletal open bite. Angle Orthod..

[28] Xiao D., Gao H., Ren Y. (2010). Craniofacial morphological characteristics of Chinese adults with normal occlusion and different skeletal divergence. Eur. J. Orthod..

[29] Rongo R., Martina S., Bucci R., Valletta R., D’Antò V., Martina R. (2019). Differences in craniofacial growth of Class II individuals from different decades: A retrospective study. Orthod. Craniofacial Res..

[30] Petrovic A., Stutzmann J., Lavergne J., Shaye R. (1991). Is it possible to modulate the growth of the human mandible with a functional appliance?. Int. J. Orthod..

[31] Franchi L., Baccetti T. (2006). Prediction of Individual Mandibular Changes Induced by Functional Jaw Orthopedics Followed by Fixed Appliances in Class II Patients. Angle Orthod..

[32] D’Antò V., Pango Madariaga A.C., Rongo R., Bucci R., Simeon V., Franchi L., Valletta R. (2019). Distribution of the condylion-gonion-menton (cogome^) angle in a population of patients from southern Italy. Dent. J..

[33] Molhave A. (1958). Sitting & standing posture in man. Ugeskr. Laeger..

[34] Solow B., Tallgren A. (1971). Natural head position in standing subjects. Acta Odontol. Scand..

[35] Siersbæk-Nielsen S., Solow B. (1982). Intra- and interexaminer variability in head posture recorded by dental auxiliaries. Am. J. Orthod..

[36] Kuitert R., Beckmann S., van Loenen M., Tuinzing B., Zentner A. (2006). Dentoalveolar compensation in subjects with vertical skeletal dysplasia. Am. J. Orthod. Dentofac. Orthop..

[37] Arriola-Guillén L.E., Flores-Mir C. (2015). Anterior maxillary dentoalveolar and skeletal cephalometric factors involved in upper incisor crown exposure in subjects with Class II and III skeletal open bite. Angle Orthod..

[38] Choi Y.J., Kim D.J., Nam J., Chung C.J., Kim K.-H. (2016). Cephalometric configuration of the occlusal plane in patients with anterior open bite. Am. J. Orthod. Dentofac. Orthop..

[39] Arriola-Guillén L.E., Flores-Mir C. (2014). Molar heights and incisor inclinations in adults with Class II and Class III skeletal open-bite malocclusions. Am. J. Orthod. Dentofac. Orthop..

[40] Kim S.-J., Kim K.-H., Yu H.-S., Baik H.-S. (2014). Dentoalveolar compensation according to skeletal discrepancy and overjet in skeletal Class III patients. Am. J. Orthod. Dentofac. Orthop..

[41] Angelieri F., Franchi L., Cevidanes L.H.S., Bueno-Silva B., McNamara J.A. (2016). Prediction of rapid maxillary expansion by assessing the maturation of the midpalatal suture on cone beam CT. Dent. Press J. Orthod..

[42] Enlow D.H., Moyers R. (1982). Handbook of Facial Growth.

[43] DeVincenzo J.P. (1991). Changes in mandibular length before, during, and after successful orthopedic correction of Class II malocclusions, using a functional appliance. Am. J. Orthod. Dentofac. Orthop..

[44] Subtelny J.D., Sakuda M. (1964). Open-bite: Diagnosis and treatment. Am. J. Orthod..

[45] Rohit K. (2018). Open bite malocclusion: An overview. J. Oral Health Craniofac. Sci..

[46] Scheffler N.R., Proffit W.R., Phillips C. (2014). Outcomes and stability in patients with anterior open bite and long anterior face height treated with temporary anchorage devices and a maxillary intrusion splint. Am. J. Orthod. Dentofac. Orthop..

